# Transient Absorption Microscopy Explores the Effect of Pt Deposition on Charge Carrier Dynamics in Individual Carbon Nitride Particles

**DOI:** 10.1002/cssc.202500203

**Published:** 2025-05-08

**Authors:** Sutripto Khasnabis, Cassandra Mesburis, Robert Godin

**Affiliations:** ^1^ Department of Chemistry The University of British Columbia 3247 University Way Kelowna BC V1V 1V7 Canada; ^2^ Clean Energy Research Center The University of British Columbia 2360 East Mall Vancouver BC V6T 1Z3 Canada; ^3^ Okanagan Institute for Biodiversity, Resilience, and Ecosystem Services The University of British Columbia Kelowna BC V1V 1V7 Canada

**Keywords:** carbon nitrides, charge transfers, heterojunctions, photocatalysis, transient absorptions

## Abstract

This study aims to spatially understand the effect of Pt deposition on slow (μs–s) carrier dynamics in carbon nitride (CN_*x*_) photocatalysts, using a homebuilt microsecond diffuse reflectance transient absorption microscopy setup. It is found that Pt deposition lengthens the overall charge half‐lives about threefold, while having a spatial preference for binding with respect to defects that induce charge trapping in the μs–s timescale. The initial trapped charge population shows a slight decrease after Pt deposition, suggesting that the more reactive and mobile charges are extracted by Pt sites, leaving behind deeply trapped and long‐lived charges. The results emphasize that spatial heterogeneity of surface cocatalysts likely plays a major role in controlling the charge carrier dynamics and activity of photocatalysts.

## Graphical abstract

Transient absorption microscopy in the microsecond and micrometer regime is used to investigate the effect of Pt on individual carbon nitride (CN*_x_*) photocatalytic particles. A pixel‐by‐pixel comparison of particles before and after Pt deposition highlights that Pt has a spatially heterogeneous impact on the charge carrier dynamics, with implications for the optimization of photocatalyst/cocatalyst interfaces.

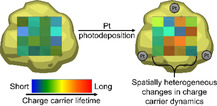

## 1 Introduction

Carbon nitride (CN_*x*_), an organic semiconductor that follows facile synthesis routes, has attracted significant attention for its potential in green H_2_ production via visible light photocatalysis.^[^ ^1^, ^2^, ^3^ ^]^ However, in its pristine form, its solar‐to‐hydrogen conversion efficiency is limited by issues such as rapid recombination rates of photogenerated charges and interlayer electrostatic barriers, which prevent efficient charge transfer to surface reaction sites.^[^ ^4^, ^5^ ^]^ To tackle these issues for enhanced applicability in the hydrogen evolution reaction (HER), deposition of noble metal nanoparticles that enable effective electron extraction has been routinely explored and serve as proton reduction sites.^[^ ^6^ ^]^ Pt nanoparticles are particularly popular, owing to specifically low overpotential for proton reduction, large work function, and are usually the most active cocatalyst among other candidates.^[^ ^6^, ^7^, ^8^, ^9^ ^]^ These properties aid in charge separation and transfer of photogenerated electrons from the bulk to the surface Pt sites and hence enhance proton reduction rates.^[^ ^10^, ^11^, ^12^, ^13^ ^]^ For instance, Li et al. demonstrated that single‐atom Pt, when used as a cocatalyst for CN_*x*_, enhanced the photocatalytic HER efficiency of CN_*x*_ to reach ≈50 times the performance of its unmodified counterpart.^[^ ^14^ ^]^

Interfacial charge transfer from CN_*x*_ to a cocatalyst site is an important step that governs and directly impacts the overall efficiency of the photocatalytic process. However, limited literature is available that explores spectroscopic understandings of interfacial charge transfer across such CN_*x*_ heterojunctions, impeding the optimization of photocatalytic systems. Specifically, there is a gap in systematically investigating charge trapping in heterojunctions with disordered systems like polymeric CN_*x*_, where charge transport behavior is complex.^[^ ^15^, ^16^ ^]^ Corp et al. investigated ultrafast transient absorption (TA) carrier dynamics across a CN_*x*_ heterojunction of bulk CN_*x*_ and an exfoliated CN_*x*_ variant.^[^ ^17^ ^]^ This report linked the electron‐transfer mechanism across the heterojunction to the increase (near‐doubling) of the photocatalytic rate of HER once the exfoliated CN_*x*_ was introduced, suggesting that oxidized chain terminations in the exfoliated CN_*x*_ are catalytically active towards HER. They also reported information about charge transfer timescales (≈ns range) for this heterojunction system. For CN_*x*_/metal heterojunctions, Tong et al. examined ultrafast TA kinetics across CN_*x*_/Pt junctions, comparing a more crystalline CN_*x*_ variant with a pristine (disordered) control. The KCl–LiCl‐treated more crystalline CN_*x*_ exhibited enhanced charge transfer to Pt, ≈59.5% within 1.6 ps, while the untreated control achieved ≈21.3% within 11.8 ps. Benisti et al. reported transient IR on the ns timescale that showed a lengthening of the CN_*x*_ transient signals from ≈100 to 150 ns when Pt was added, attributed to shallow trapping of electrons in Pt sites and holes nearby, linked to a threefold enhancement in the rate of water oxidation.^[^ ^18^ ^]^ These examples highlight how ns and shorter timescale spectroscopic examination of charges across heterojunctions are related to photocatalytic performance. However, the reported charge transfer efficiencies to Pt ≪ 100% indicate that a significant fraction of charges either recombines or potentially survives to longer times (μs–s) via multiple trapping and release (MTR).^[^ ^19^, ^20^ ^]^

While these examples explore the sub‐μs timescales, we note that events such as charge trapping, diffusion, and extraction on the μs–s timescales are crucial for bridging the full story of HER activity. We emphasize that the μs–s timescale is of particular importance to HER since proton reduction takes place approximately in the millisecond timescale.^[^ ^21^, ^22^ ^]^ However, spectroscopic investigations of these slower charge carrier transport processes are underrepresented in current literature.^[^ ^23^, ^24^ ^]^ Additionally, we note that CN_*x*_ suspensions are routinely used for transmission TA measurements, which necessitate drastic size reduction for achieving suspension stability.

We introduce a different approach using our home‐built μs‐diffuse reflectance TA microscope (μs‐DR‐TAM) to examine the charge carrier dynamics of single CN_*x*_ heterojunction particles with size ranges that are relevant to HER conditions.^[^ ^3^, ^25^, ^26^ ^]^ We have applied this technique to pristine CN_*x*_ particles in our previous work, where we identified micrometer‐sized spatial defects that control the charge carrier dynamics in the μs–s timescale.^[^ ^27^ ^]^ In the current study, we aim to address the knowledge gap of the effect of Pt deposition on these long‐lived charges in CN_*x*_ photocatalysts to learn more about the factors that impact photocatalytic activity.

**Figure 1:**
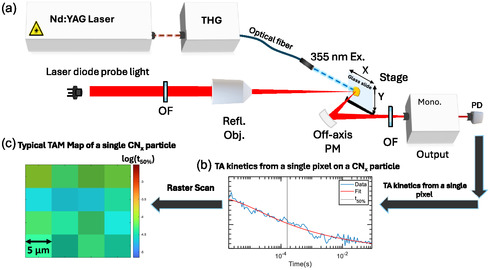
a) Schematic demonstrating the μs‐DR‐TAM setup used for our experiments using a laser diode light source (820 nm). b) TA signal from a single CN_*x*_ particle measured and fitted to a power law decay. c) Typical TAM map performed on a single particle (scale bar‐5 μm). Abbreviations: THG, third‐harmonic generator; OF, optical filter; off‐axis PM, off‐axis parabolic mirror; mono., monochromator; PD, photodetector; Refl. Obj, reflective objective.

We used our homebuilt μs‐DR‐TAM setup (**Figure** **1**a) to measure the spatial effect of Pt deposition on the TA behavior of single CN_*x*_ particles. The CN_*x*_ particles were prepared from the typical thermal polymerization route from dicyandiamide (DCDA) precursor, in line with our previous work^[^ ^27^ ^]^ and as described in Section 1.2, Supporting Information. The particles were excited by a 355 nm laser, and the charge carrier dynamics were measured with an 820 nm probe beam focused to 5 μm full width at half maximum (FWHM; see Section 1.3 and 1.4, Supporting Information). At this wavelength, the monitored TA signal is exclusively attributed to trapped electrons in CN_*x*_, as previous reports of transient absorption spectroscopy (TAS) spectra with and without Pt have shown the same spectral features.^[^ ^2^, ^28^, ^29^, ^30^ ^]^ We have shown that these types of decays are independent of the presence of oxygen.^[^ ^27^ ^]^ As the HER activity from single CN_*x*_/Pt particles cannot be determined with our setup (see Section 2, Supporting Information), we prepared CN_*x*_ films for quantification of HER activities by varying the photodeposition time of Pt and determining the optimal Pt loading for HER activity of these particles in a representative manner. These results (Figure S1, Supporting Information) revealed that 60 min of Pt deposition gave the optimum HER rates.

We conducted scanning transmission electron microscopy/energy‐dispersive X‐ray spectroscopy to confirm Pt deposition on the CN_*x*_ particles (Section 3, Figure S2, S3, Supporting Information). Measured Pt densities were 2.6 particles/nm^2^ (5 min Pt/CN_*x*_) and 3.3 particles/nm^2^ (60 min Pt/CN_*x*_), indicating that a longer deposition time increases the number of particles on the surface. We also observed a slight increase in the size of the Pt particles at the longer deposition time (Figure S4, Supporting Information). The particle density measured by TEM corresponds to a spatially averaged effect over ≈50 million and 65 million particles within the 5 μm FWHM probe for the TAM measurements, respectively. Thus, on the micrometer scale, the spatial TAM maps will not show identifiable TA features characteristic of local Pt deposition since the probed area will average the response of many millions of sub‐nm‐diameter Pt particles, which are evenly distributed on the surface of CN_*x*_.

We sought to explore the spatial effect of Pt on the charge carrier dynamics of single particles of CN_*x*_ with the optimal Pt loading (CN_*x*_/Pt_60min_). However, we determined that significant structural changes took place during sample irradiation and handling, and we could not acquire data on the same region of CN_*x*_ particles before and after 60 min of Pt deposition (Section 4, Figure S5, S6, Supporting Information). We thus acquired TAM maps on the same particles before and after Pt deposition, but did not perform pixel‐to‐pixel comparisons. The overall changes in TA behavior were investigated statistically by considering each pixel independently. We measured 4 × 4 pixels TAM maps on seven particles before and after 60 min of Pt deposition. The probe spot was raster scanned to realize 20 × 20 μm TAM maps with 5 μm step sizes. The resulting TA trace (0.1 s in total length) from a single pixel is shown in Figure 1b. The decay is mathematically modeled as a power law according to the MTR model of charge recombination^[^ ^19^, ^20^ ^]^

(1) $$\begin{array}{c} \text{Absorptance= }\frac{{\text{Abs}}_{0}}{\;{\left(\text{1}+bt\right)}^{\alpha }\;}{+\text{Abs}}_{\infty } \end{array}$$

Here, *t* refers to time, Abs_0_ is the absorptance extrapolated to time = 0, Abs_∞_ is the absorptance offset at infinite time, *b* is approximately the inverse of the decay onset time, and *α* is a measure of the energetic distribution of trap states with values ranging between 0 and 1.^[^ ^20^ ^]^ From the fits two parameters were extracted: 1) the initial signal amplitude, absorptance(*t*_0_), where *t*_0_ is chosen as 1.5 μs since it is the earliest time where the data have a satisfactory signal‐to‐noise ratio and 2) the half‐life (*t*_50%_) values, the time where the initial amplitude at 1.5 μs decreases by half, providing information on the decay kinetics of the charges.^[^ ^28^ ^]^

**Figure 2:**
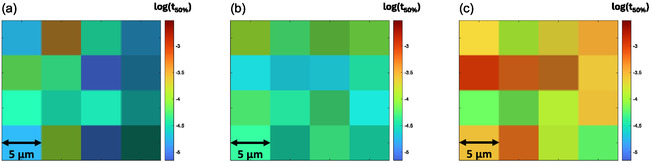
CN_*x*_ single‐particle TAM map examples revealing a general shift in pixel colors across different particles and conditions because of altered half‐lives (*t*_50%_ values). a) Particle B before Pt deposition, b) Particle A before Pt deposition, and c) Particle A after 60 min of Pt deposition.

The sizes (projected areas) of particles measured varied from ≈2 to 15 × 10^4^ μm^2^, corresponding to diameters of a few hundred micrometers. The color scale on a TAM map (Figure 1c, **Figure** **2**) represents the logarithm of *t*_50%_ values, ranging between 10^−2^ s (10 ms; red) and 10^−5.25^ s (5.6 μs; blue). These false color images additionally present the absorptance(*t*_0_) as brightness, where darker/brighter pixels have lower/higher values of absorptance(*t*_0_) (Figure S7, Supporting Information). Figure 2 shows three representative examples of TAM maps: Figure 2a is dominated by blue shades, indicating more regions with shorter half‐lives, Figure 2b is cyan‐green with half‐life values around the center of the color scale, and Figure 2c is dominated by orange–red shades, which point to longer half‐lives.

**Figure 3:**
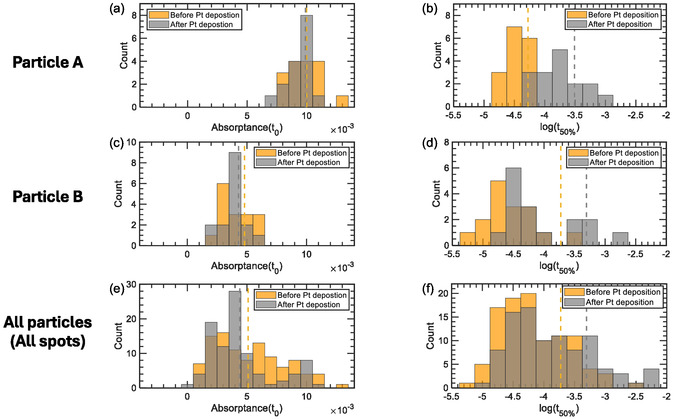
Histograms depicting the frequency distributions of the parameters from the TA decay data for particles before and after 60 min of Pt deposition. Particle A results for a) the absorptance(*t*_0_) and b) the *t*_50%_ parameters. Particle B results for c) the absorptance(*t*_0_) and d) the *t*_50%_ parameters. Results across all particles (all spots) for e) the absorptance(*t*_0_) and f) the *t*_50%_ parameters. The dashed lines represent the baseline values (orange) and the predicted outcome (gray) from the GLMM fit results before and after Pt treatment, respectively.

The measurements before and after 60 min of Pt deposition on seven particles yielded ≈100 pixels for analysis under each condition. Around 15% of the total TA data were excluded due to unsatisfactory signal‐to‐noise ratios (Figure S10, Supporting Information). In our previous study,^[^ ^27^ ^]^ we concluded that two spatially distinct defects influence the absorptance(*t*_0_) and *t*_50%_ independently with varying densities across CN_*x*_ particles. In agreement with this conclusion, the pixel‐wise log(*t*_50%_) versus absorptance(*t*_0_) data for individual particles shows no correlation (Figure S18, Supporting Information). However, in this study, we do observe an overall color shift in the maps from largely blue to orange–red following Pt deposition, indicating a lengthening of the *t*_50%_. Consequently, to draw statistically robust conclusions, we analyzed the effect of Pt deposition on our measured parameters using a generalized linear mixed model (GLMM), which allows consideration of non‐normal data distributions and accounts for random effects such as particle‐to‐particle TA heterogeneity (Section 7, Supporting Information).^[^ ^31^, ^32^, ^33^ ^]^ In this framework, we define the baseline values as the parameters before Pt deposition, B(*t*_50%_) and B(Abs‐t_0_), while the predicted outcomes, P(*t*_50%_) and P(Abs‐t_0_) are the estimated parameter values after Pt deposition, incorporating the predictor effect (Pt deposition) as a multiplicative effect (Table S1, Supporting Information). **Figure** **3** shows histograms of the parameters extracted from fits of the decays for particles A and B as specific examples (Figure 3a–d; Figure S8, Supporting Information) and for all seven particles (Figure 3e,f).

For particle A, the B(*t*_50%_) value changes from 53 μs before Pt to P(*t*_50%_) = 309 μs after 60 min of Pt deposition (Table S1, Supporting Information). For particle B, the B(*t*_50%_), which is 68 μs, results in a P(*t*_50%_) = 366 μs. When considering all particles, the B(*t*_50%_) = 188 μs before Pt changes to P(*t*_50%_) = 495 μs after Pt deposition.

In contrast, the absorptance(*t*_0_) values tend to decrease by about 10% after Pt deposition. The decrease is from the baseline value B(Abs‐t_0_) = 0.0103 to the predicted P(Abs‐t_0_) = 0.0099 for particle A, from 0.0048 to 0.0043 for particle B, and from 0.0051 to 0.0044 for all particles. A considerable fraction of the α parameter was overestimated to yield *α* = 1 during the fitting of TA decays. GLMM fits (Table S2, Supporting Information), excluding the *α* = 1 values, were performed, but we refrained from drawing in‐depth inferences. The *α* distributions (Figure S13, Supporting Information) are similar before and after Pt, suggesting that the energetic distributions of trap states aren't significantly influenced by the presence of Pt.

After inspecting the statistical changes in the TA parameters from the optimally HER‐performing Pt‐loaded particles, we sought to investigate the spatial effects of Pt deposition to leverage the full capability of our setup that enables spatial correlation at the micrometer level. Since we have evidence that the local environment controls the lifetime and is resolvable by the 5 μm FWHM probe,^[^ ^27^ ^]^ spatially correlating the *t*_50%_ parameter before and after Pt deposition can illustrate how Pt affects environments with varying *t*_50%_ values. As 60 min of Pt deposition disrupted the CN_*x*_ particle structure (Figure S5, Supporting Information), we proceeded to explore the effect of Pt using the lowest deposition time (5 min). With 5 min of Pt deposition, the initial particle shape was retained, enabling pixel‐by‐pixel spatial correlation before and after Pt deposition (Figure S6, Supporting Information).

Six new particles (particles C, D, and E as examples) were examined before and after 5 min of Pt deposition by TAM mapping (Figure S6, Supporting Information). The results from these particles are represented in Figure S14, S15, Supporting Information, and confirm that the same trends are observed for both 5 and 60 min of Pt deposition. The predicted outcome for *t*_50%_ considering all 96 pixels of the six particles increased threefold from the baseline value after 5 min of Pt deposition (B(*t*_50%_) = 66 μs and P(*t*_50%_) = 193 μs), and the absorptance(*t*_0_) decreases by 10%, from B(Abs‐t_0_) = 0.0060 to P(Abs‐t_0_) = 0.0054 (Table S3, Supporting Information).

A report by Lau et al. provides evidence of chemical groups (e.g., urea moieties on a modified CN_*x*_) facilitating metal–support interactions with Pt that resulted in strong electronic coupling, promoted charge transfer, and improved photocatalytic hydrogen evolution.^[^ ^34^, ^35^ ^]^ In this context, we examined whether Pt affects the charge carrier dynamics of all pixels uniformly or if there is a more significant effect of Pt tied to higher or lower *t*_50%_ values and their corresponding defects. We performed a quartile analysis (see Section 9, Figure S16, Supporting Information) based on the *t*_50%_ values from the TAM maps of the six particles before 5 min of Pt deposition (**Table** **1**). For each quartile, we compared the change in *t*_50%_ parameter using a GLMM model for the same region on the CN_*x*_ particle before and after 5 min of Pt deposition (i.e., the same pixel on the TAM maps before and after Pt). All pixels (100%) of quartile 1, the subset of pixels with the lowest *t*_50%_ before Pt deposition, showed an increase in *t*_50%_ values after Pt deposition. For the next quartiles, a decreasing number of pixels showed an increase in *t*_50%_, with quartiles 2, 3, and 4 exhibiting an increase in 96, 79, and 50% of pixels, respectively.

**Table 1:** Quartile analysis of the *t*_50%_ values of particles before and after 5 min of Pt deposition.

	Pixels with longer *t*_50%_ after Pt	Baseline *t*_50%_ value before Pt	Predicted *t*_50%_ value after Pt	Ratio of *t*_50%_ before and after Pt
Quart.1	100%	19 μs	203 μs	10.5
Quart.2	96%	31 μs	99 μs	3.2
Quart.3	79%	49 μs	292 μs	5.9
Quart.4	50%	190 μs	212 μs	1.1

The quartile analysis reveals that regions of CN_*x*_ particles that have short *t*_50%_'s are more likely to show large increases in *t*_50%_ when Pt is deposited. Notably, the longest P(*t*_50%_) seen post‐Pt deposition is for quartile 3, and quartile 1 has the second longest P(*t*_50%_), ruling out that the larger increases in *t*_50%_ are solely due to the shorter initial *t*_50%_. These results indicate that the effect of Pt is not uniform, presumably due to heterogeneous accumulation of Pt around different chemical environments. Since these quartiles form subsets of the full range of *t*_50%_ values, which can be broadly affiliated with different defect types and/or different defect densities, the uneven changes across quartiles indicate that Pt binding is sensitive to the local chemical environment.

**Figure 4:**
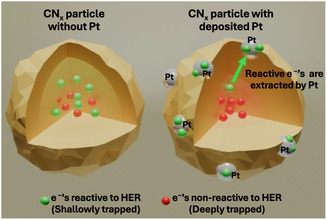
Schematic showing the effect of Pt deposition on reactive and nonreactive charges (toward HER) in a single CN_*x*_ particle.

Across both datasets for 5 and 60 min of Pt deposition, values of absorptance(*t*_0_) shift to lower values after Pt deposition, consistent with electrons being extracted by Pt. This aligns with the concept of Pt acting as an electron sink, which electronically refers to the formation of a Schottky junction, facilitating the energetically downhill movement of reactive electrons, resulting in spatial separation of charges with longer lifetimes.^[^ ^6^ ^]^ This extended lifetime is beneficial for consequent electron transfer to species like H^+^ for HER, as exemplified in systems like TiO_2_ photocatalysts.^[^ ^36^, ^37^ ^]^ These sites extract reactive electrons, making them available for HER under reaction conditions. We link the observed increase in the *t*_50%_ parameter after Pt deposition to the longer lifetimes of the remaining deeply trapped charges after the more mobile and active charges in shallow traps have been extracted by Pt. The extraction of the mobile, reactive charges and the remnant long‐lived reactive charges are schematically shown in **Figure** **4**.

To conclude, we acquired and compared TAM maps of CN_*x*_ particles before and after Pt deposition using a μs‐DR‐TAM setup to explore spatial changes in the charge carrier dynamics. We find that spatial features due to Pt deposition cannot be resolved on the micrometer scale when considering the charge carrier dynamics in the μs–ms timescales. However, following Pt deposition, the overall half‐lives of trapped charges in these timescales become longer. Additionally, from spatially correlated studies of individual CN_*x*_ particles, we conclude that Pt has a larger influence on the charge carrier dynamics in regions that have shorter *t*_50%_ before Pt deposition. The spatial heterogeneity in the *t*_50%_ increase is potentially linked to the chemical nature of the spatial defects that dictate the half‐lives of charges. Additionally, the initial density of trapped charges decreases by 10% following Pt deposition. Taken together with the increased *t*_50%_, these results suggest that shallowly trapped charges (more mobile and reactive) are selectively extracted by Pt sites, leaving behind deeply trapped charges with longer half‐lives. These measurements demonstrate how μs‐DR‐TAM can be leveraged to gain a better understanding of the charge carrier dynamics in heterojunctions of disordered semiconductors.

## Conflict of Interest

The authors declare no conflict of interest.

## Supporting information

Supplementary Material

## Data Availability

Data represented in the figures of the main text are available in the Federated Research Data Repository (FRDR) under DOI: 10.20383/103.01170.
