# Supplementary material for: Transient Absorption Microscopy Explores the Effect of Pt Deposition on Charge Carrier Dynamics in Individual Carbon Nitride Particles
Source: ChemSusChem. 2025 May 8;18(13):e202500203. doi: 10.1002/cssc.202500203 (PMC12232084; doi:10.1002/cssc.202500203)
Supplement: Supplementary file 1 — Supplementary Material [file CSSC-18-e202500203-s001.pdf]

Supporting information

Transient Absorption Microscopy Explores the Effect of Pt Deposition on Charge Carrier Dynamics in Individual Carbon Nitride Particles

Sutripto Khasnabis[a], Cassandra Mesburis[a] and Robert Godin[a,b,c]*

[a] Department of Chemistry, The University of British Columbia, 3247 University Way, Kelowna, BC, V1V 1V7, Canada

[b] Clean Energy Research Center, University of British Columbia, 2360 East Mall, Vancouver, BC, V6T 1Z3, Canada

[c] Okanagan Institute for Biodiversity, Resilience, and Ecosystem Services, University of British Columbia, Kelowna, BC, Canada

Corresponding author email: robert.godin@ubc.ca

Table of Contents

1. Materials and Methods S4
1.1 Materials S4
1.2 Synthesis of CN_x particles S4
1.3 Slide preparation and Pt deposition S4
1.4 Experimental Setup for Transient Absorption Microscopy (TAM) S5
2. Hydrogen evolution activity (HER) for different Pt loadings on CN_x films S7
Figure S1.. .... S7
3. Scanning transmission electron microscopy/Energy-dispersive X-ray spectroscopy (STEM/EDS) results showing Pt deposition S9
Figure S2. .... S9
Figure S3. .... S9
Figure S4.. .... S10
4. Diffuse-reflectance light intensity maps of particles for spatial correlation S10
Figure S5. .... S11
Figure S6. .... S12
5. Typical TAM Maps showing the color scheme and parameters, TAM maps for particles A-E before and after Pt deposition........ S13
Figure S7. .... S13
Figure S8. .... S14
Figure S9. .... S15
6. Example of rejected TA trace S16
Figure S10. .... S16
7. Generalized Linear Mixed Model to predict effect of Pt on TA parameters in CN_x........ S17
Figure S11. .... S18
Figure S12. .... S18
8. Histograms of α parameter and GLMM fit results for α, absorptance(t_0) and $t_{50\%}$ from TA kinetics before and after Pt deposition........ S19
Figure S13.. .... S19
Table S1........ S19
Table S2......... S20
9. Data from spatially correlated TAM measurements before and after 5 minutes of Pt deposition (6 particles) S21
Figure S14. .... S21
Figure S15. .... S21

Table S3. .... S22
Figure S16. .... S23
10. TA decay trace fitting and determination of the parameters S24
Figure S17. .... S24
11. Correlation between $t_{50\%}$ vs. Absorptance(t_0) including all pixels of the TAM data collected from all bare CN_x particles (from the 60-minute Pt deposition data set) S25
Figure S18. .... S25
References S26

1. Materials and Methods

1.1 We declare that no unexpected or unusually high safety hazards were encountered.Materials

Acetonitrile (ACN) (99.8%), chloroplatinic acid hexahydrate ($H_2PtCl_6 \cdot 6H_2O$), dicyandiamide (DCDA; 99%), triethanolamine (99%) and hydrochloric acid (37%) were purchased from Sigma-Aldrich. Sodium hydroxide (NaOH) was obtained from VWR Chemicals and potassium hydroxide (KOH) from TCI. All chemicals used were of reagent grade or higher.

1.2 Synthesis of CN_x particles

CN_x particles used in the experiments were synthesized using dicyandiamide (DCDA), which was heated to 550 °C for 4 h, at a ramp rate of 2 °C min^{-1} in a covered crucible.[1,2] The sample was naturally cooled in the furnace. The resulting mass was then repeatedly washed with the following solvents, in this order: (1) water, (2) 1 M KOH (aq.) (3) 1 M HCl (aq.) (4) acetonitrile, (5) water, (6) 1 M KOH (aq.) (7) 1 M HCl (aq.) (8) water. Each wash included sonicating the product with the solvent for about 10 minutes, followed by centrifugation and removal of the washing solvent. The washed sample was dried in a heating oven at 80 °C, overnight. The product was then ground using a mortar and pestle to generate a powder, form which single particles were isolated (picked with tweezers).

1.3 Slide preparation and Pt deposition

The single particles of CN_x were stuck on a cleaned glass slide (approximately 2.5 cm x 2.5 cm) using a water-resistant epoxy (System Three Submarine Underwater Epoxy).

For Pt deposition, the glass slide containing the particles was immersed in 10 mL aqueous solution with triethanolamine (TEOA; 10% v/v) and chloroplatinic acid (0.5 mM) in a crystallizing dish. The solution was purged for 15 minutes with argon and then illuminated using AM 1.5 Solar radiation generated by a Solar Simulator (Sciencetech SciSun Series Arc Lamp System), with constant argon flow. The slide was immersed with the particles facing up (towards the light) and illuminated for varying amounts of time to drive Pt deposition.

1.4 Experimental Setup for Transient Absorption Microscopy (TAM)

A home-built setup was used for Transient Absorption Microscopy (TAM) measurements with a continuous wave (CW) laser diode probe. Optical bandpass filters (800FS00-25, Andover Corporation; ~ 800 nm centre, ~ 100 nm bandwidth) were used to further optically filter the light from laser scatter and undesirable emissions contributions. The laser diode used was L820P100 (Thorlabs, Inc.) outputting 820 nm. The laser diode was regulated using a low-noise laser diode controller (LDC200C, Thorlabs, Inc.) and the temperature control was achieved using a temperature-controlled mount (LDM56, Thorlabs, Inc.) using a TEC controller (TED200C, Thorlabs, Inc.). The probe light was focused on the sample using a reflective objective: LMM-15X-P01 (Thorlabs, Inc. Numerical aperture (NA) - 0.30). The diffuse reflected light was collected by an Oriel Cornerstone monochromator (Newport Corporation) for selecting the probe wavelength. From the monochromator, the probe light was projected onto a mounted Si photodiode (Thorlabs, Inc.). The resulting transient photocurrent signal was amplified, converted to voltage, and filtered by custom analog electronics (low pass filter cut-off ∼ 1.5 µs, high pass filter cut-off ∼ 1 s). This signal was recorded by an oscilloscope (National Instruments). A Surelite II Continuum Nd:YAG laser (1064 nm fundamental output) was used for photoexcitation, frequency tripled to generate laser pulses at 355 nm (pulse width < 10 ns). A 300 µJ cm^{-2} laser fluence used for the measurements. The excitation pulse area in the sample plane was much larger than the focussed probe beam, about 0.25 cm^2. For each measurement, the signal was averaged over 128 scans. The time resolution of the measurements were 64 ns and the total acquisition lengths were 0.1 s. The response time of the setup is typically on the order of 1 – 10 µs.

Data were handled with custom-written MATLAB scripts (https://github.com/SolarSpec/SpectraBuilder, https://github.com/SolarSpec/TAMviewer). A typical measurement consisted of the sample (CN_x particles on glass slide using water-resistant epoxy) mounted on a 2D nanopositioning stage consisting of two linear actuators (CONEX-TRB25CC, Newport Corporation). The stage was made to align to the focal plane of the probe beam in a direction parallel to the probe with a micrometre drive. The stage was translated in the X and Y directions, in a plane perpendicular to the direction of the probe light. The data acquisition was controlled by a custom software written in the LabVIEW environment to achieve the raster scan for TAM maps.

2. Hydrogen evolution activity (HER) for different Pt loadings on CN_x films

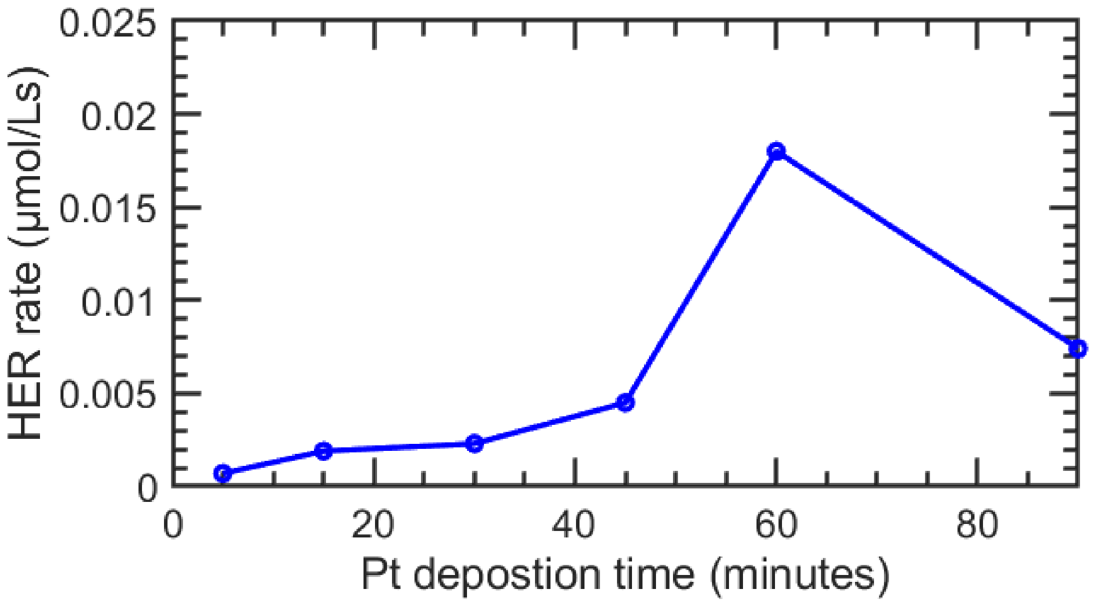

Figure S1. Hydrogen evolution rate (HER) rates for different Pt loadings on CN_x films.

CN_x films (2 cm x 2 cm) were prepared by applying compact CN_x powder on a layer of uncured water-resistant epoxy layer (System Three Submarine Underwater Epoxy) on a cleaned glass slide substrate. These films were left to cure for about 4 hours. Excess CN_x from the top layers was dusted off and Pt was deposited using solar simulator illumination as explained in section 1.3. By varying the time of deposition, different loadings of Pt were achieved. The H_2 production rate was measured by inserting a Clark-electrode hydrogen microsensor (Unisense) into the solution cuvette, in which the CN_x film was irradiated with a 405 nm LED, with an average power density of ~10 mW cm^{-2}. The current generated in the electrode was converted to a voltage readout (Unisense Microsensor Multimeter) and a two-point calibration curve (0 and 20 μM H_2 dissolved in water) was used to convert the signal to H_2 concentration in μM. From these measurements we see that 60-minute deposition time achieves the highest HER rate and is the most optimized deposition time.

The first set of TAM measurements were performed on particles A, B and 5 other particles (for significant statistical data) as explained in the main text before and after 60 minutes of Pt deposition to study the effect of Pt deposition at loadings corresponding to optimal HER

activity. Since the shapes of the particles changed drastically during Pt deposition for a prolonged time (explained in section 4), we additionally investigated a new set of particles from the same batch (C, D, E; 6 in total) with the shortest Pt deposition time (5 minutes) which did not cause structural changes to the particles.

3. Scanning transmission electron microscopy/Energy-dispersive X-ray spectroscopy (STEM/EDS) results showing Pt deposition

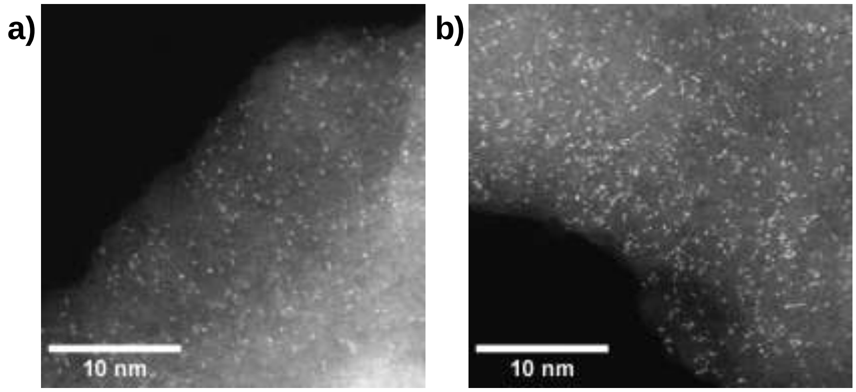

Figure S2. Dark Field STEM images showing Pt as bright spots in the 5 min (a) and 60 min (b) Pt deposited CN_x.

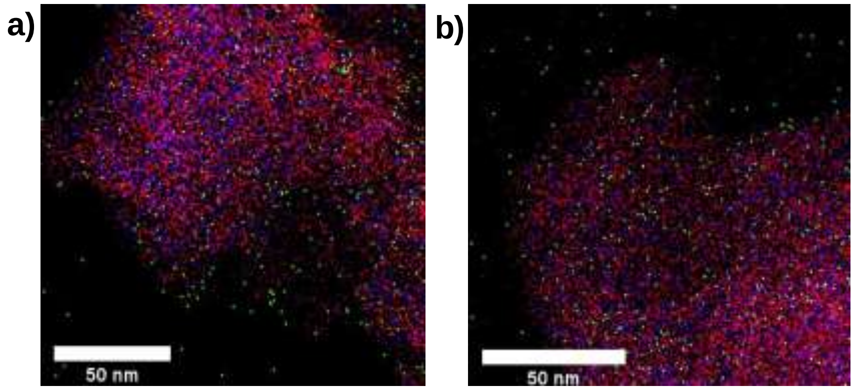

Figure S3. The STEM-EDS maps show C and N in red and blue respectively and Pt is shown in green for the 5 min (a) and 60 min (b) Pt deposited CN_x samples. The images show that Pt is largely concentrated inside the CN_x area, confirming deposition of Pt in both cases.

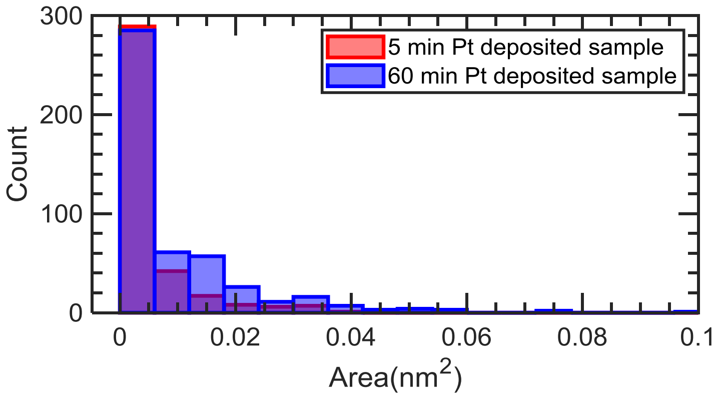

Figure S4. Histogram showing projected areas (sizes) of Pt nanoparticles after 5 and 60 minutes of Pt photodeposition on the CN_x samples calculated from the Dark Field STEM images.

4. Diffuse-reflectance light intensity maps of particles for spatial correlation

Diffuse-reflectance light intensity maps were produced by raster scanning the focussed probe light over a CN_x particle to obtain a map with 25 μm steps. This was done to capture the shape of the particles before and after Pt deposition. The relative signal intensity represents the diffuse reflectance light level at each pixel while scanning over a particle.

Firstly, light level maps were performed on a single CN_x particle before and after 60 minutes of Pt deposition (**Figure S5a and b**, respectively). These maps reveal that the initial shape of the particle is not retained after an extended deposition time of 60 minutes.

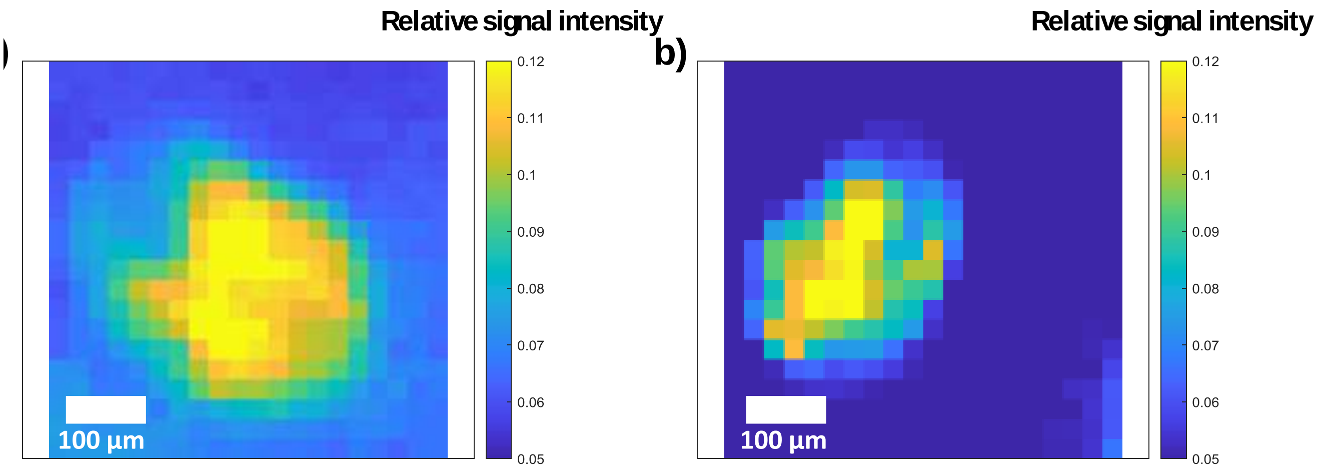

Figure S5. Diffuse reflection light level maps of a CN_x particle a) before 60 minutes of Pt deposition b) after 60 minutes of Pt deposition.

On obtaining maps on a single CN_x particle before and after 5 minutes of Pt deposition (**Figure S6a** and **b** respectively), we see that the shape is well retained after Pt deposition. **Figure S6c** depicts the maps plotted together for easier visual comparison of the chosen pixel. The pixel chosen for a 20 μm x 20 μm TAM map is indicated as a black square on all the images. Six particles (including particles C, D and E) were measured in a similar fashion for spatially correlated TAM studies before and after Pt deposition. Slight differences in the shapes (noticeable at the edges) originate from variations in the focussed beam caused due to re-acquisition of the maps after Pt deposition (requiring repositioning of the glass slide and particle on the sample stage).

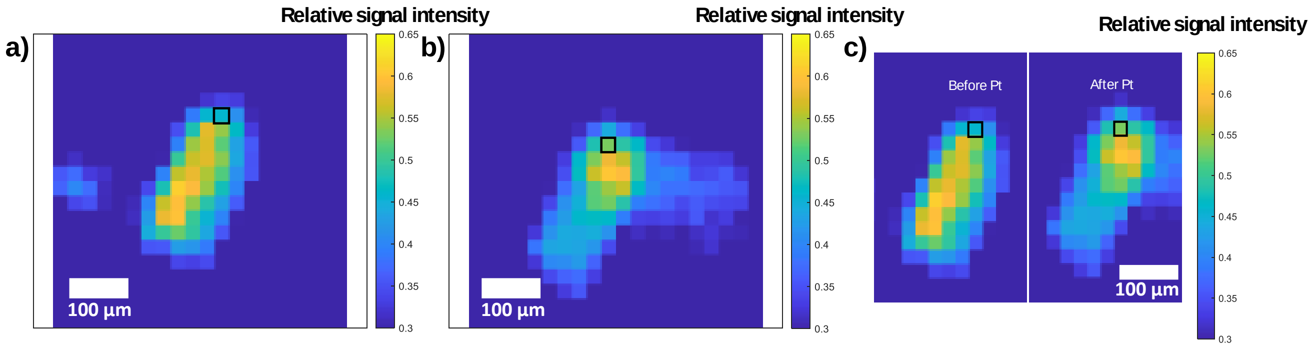

Figure S6. Diffuse reflection light level maps of CN_x particle C a) before 5 minutes of Pt deposition and b) after 5 minutes of Pt deposition. c) maps plotted together for clearer visual inspection of chosen area (marked in black) for TAM measurements.

5. Typical TAM Maps showing the color scheme and parameters, TAM maps for particles A-E before and after Pt deposition

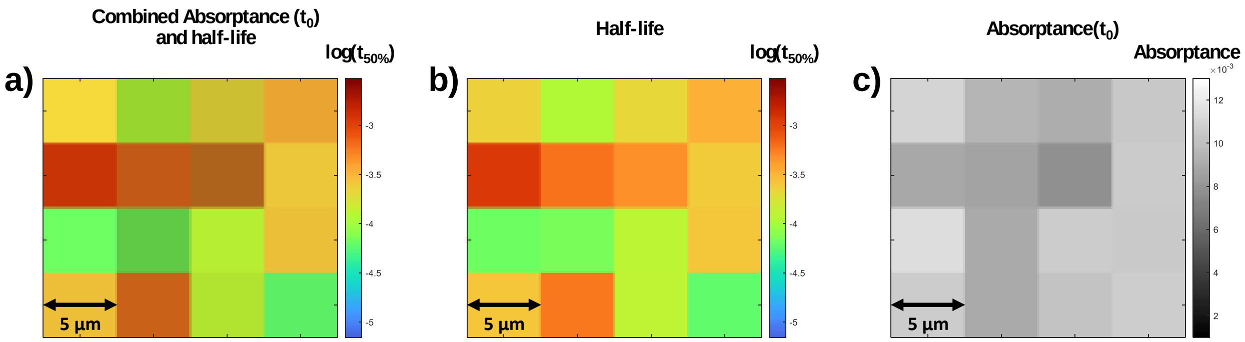

Figure S7. TAM map construction: a) Combined false color images representing absorptance(t_0) as brightness and $t_{50\%}$ as color for particle A after 60 minutes of Pt deposition. b) $t_{50\%}$ values represented using blue to red colors. Blue to red colour scale represents short to long $t_{50\%}$ values in a logarithmic scale. c) Grayscale image showing variations in absorptance(t_0).

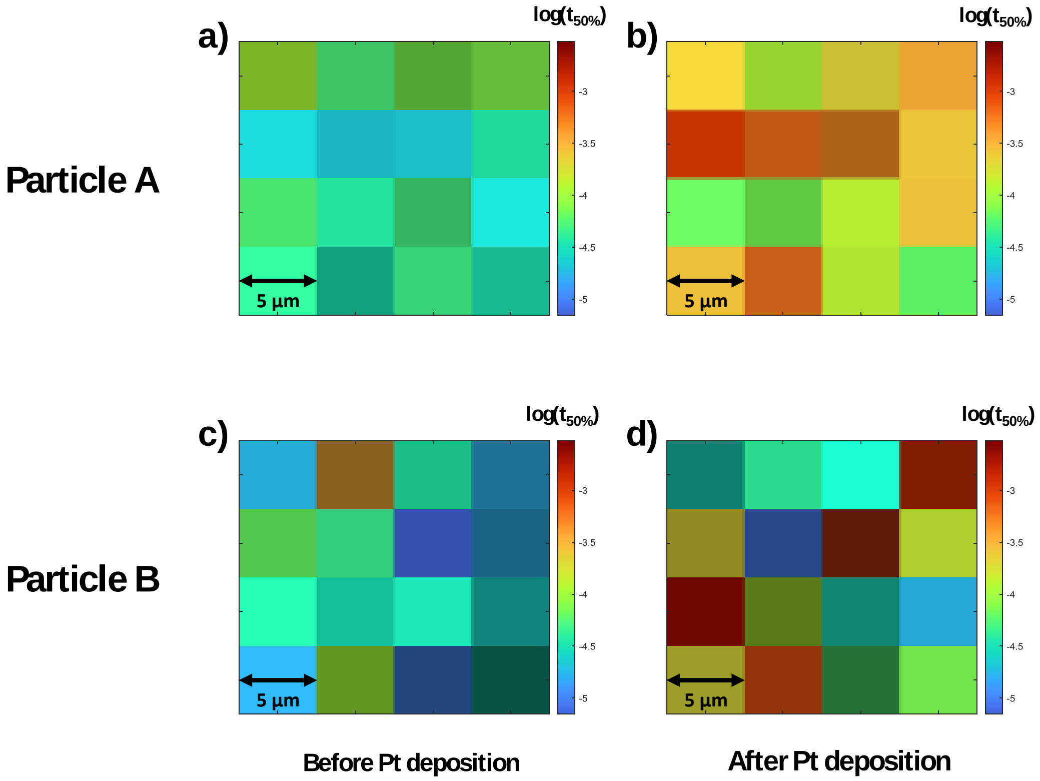

Figure S8. TAM maps of particles A and B before (a and c) and after 60 minutes of Pt deposition (b and d), respectively.

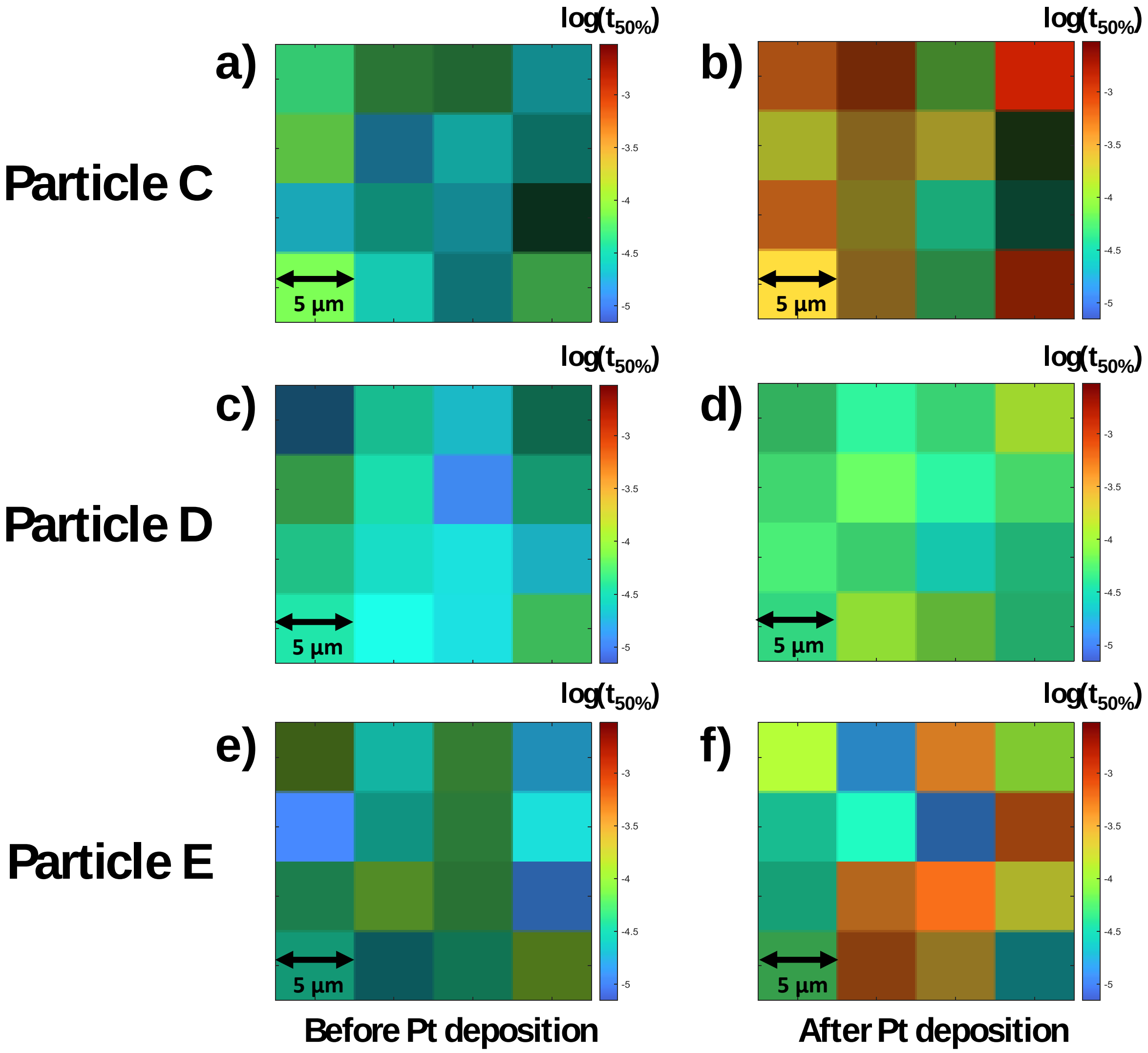

Figure S9. TAM maps taken on the same spatial spot on particles before (a, c and e) and after 5 minutes of Pt deposition (b, d and f), respectively.

6. Example of rejected TA trace

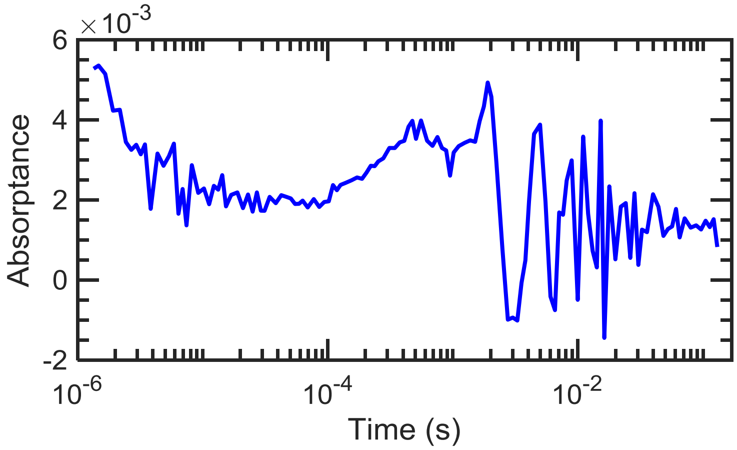

.

Figure S10. Example of a TA kinetic trace from a rejected pixel. The data had very low signal-to-noise and extracting parameters from fitting would yield unreliable results.

7. Generalized Linear Mixed Model to predict effect of Pt on TA parameters in CN_x

In this study, we measured TA parameters, $t_{50\%}$ and Abs(t_0), which exhibited a non-normal distribution, with a positive skew and positive values. We employed a Generalized Linear Mixed Model (GLMM) with a Gamma distribution and a log link function.[3]

Given the skewness of the data which approximated a Gamma function, we applied a log link function to ensure a multiplicative relationship between predictor (Pt deposition) and the response (log(E)).

In our GLMM framework, the response variable (E), $t_{50\%}$ and Abs(t_0), is modeled using a log-link:

$$\log(\mathrm{E}) = \beta_0 + (\beta_1 \times \mathrm{Pt}) + (\text{random effects from particle} - \text{to} - \text{particle variability}) \#(1)$$

Here, log(E) is the log transformed response, β_0 is the baseline intercept, β_1 is the predictor coefficient (for Pt deposition) and Pt is the predictor variable.

The initial case where there is no Pt deposition (Pt = 0), E = B($t_{50\%}$) or B(Abs-t_0) in the case of $t_{50\%}$ and Abs(t_0) respectively, where B denotes ‘Baseline value’

After Pt deposition (Pt = 1), E = P($t_{50\%}$) or P(Abs-t_0) in the case of $t_{50\%}$ and Abs(t_0) respectively, where P denotes ‘Predicted value of the model’.

We have defined a random effect in our dataset that incorporates particle-to-particle TA heterogeneity. Typically, random effects represent individual or subject-level variations that account for the heterogeneity or variability in observations.[4]

The GLMM model explained was used to model the data and quantify the effect of Pt deposition for the 5 min and 60 min deposited samples.

For diagnostics of the GLMM fit results, we plotted quantile-quantile plots (QQ plots) for the residuals which show that the residuals followed an approximately linear trend, confirming that

the model provided a good fit for the data. Sample QQ plots are shown for the parameters 5 min Pt deposited samples (**Figure S11**) which show high linearity. Across the two sets (5 min CN_x/Pt and 60 min CN_x/Pt) conisdering all particles, we find that p-values of the detemined intercepts range in the order of ~10^{-100} and the predictors (Pt) in the range between ~10^{-8} and 10^{-2} , which shows that the effect of the predictor is highly statistically significant (p-value< 0.05). We also investigated residuals vs. fitted values and residuals vs. actual values, which should ideally show random scatter around the zero line. Plots for these cases are shown in **Figure S12a** and **b**, respectively**,** which show no particular trend or shape of the scattered points.

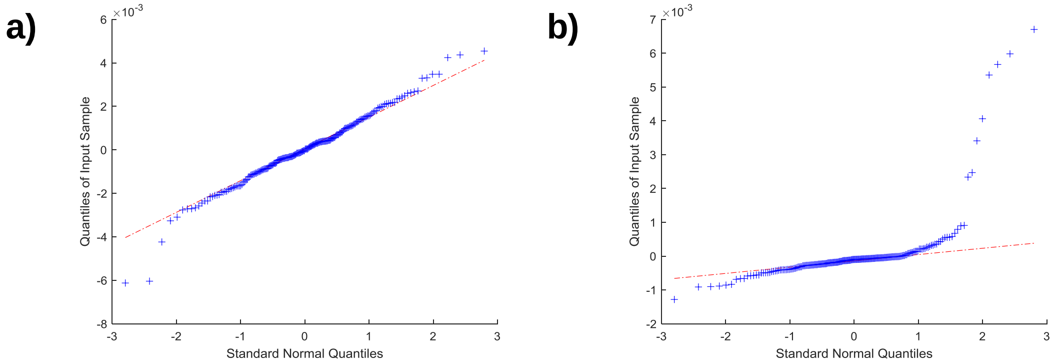

Figure S11. QQ plots for (a) absorptance(t_0) and (b) $t_{50\%}$ parameters derived from the 5 minutes Pt deposited samples (all particles).

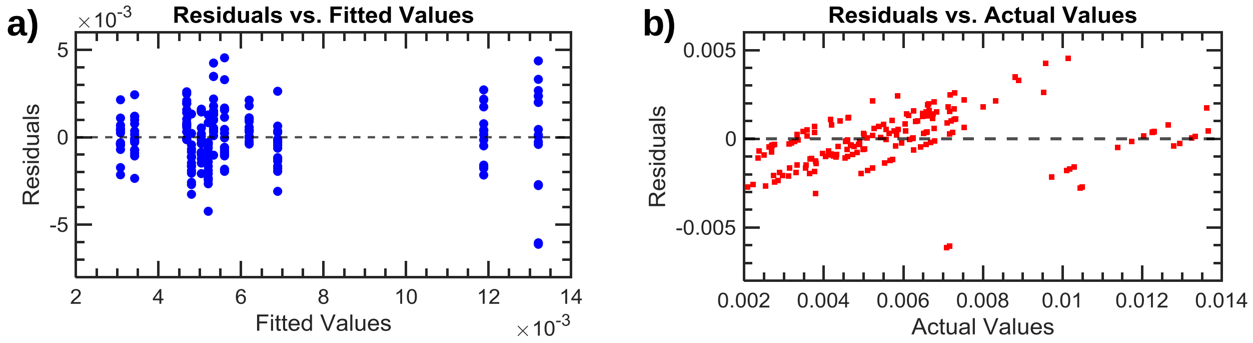

Figure S12. Examples of (a) residuals vs fitted values and (b) residuals vs. actual values plots from GLMM fit results for the absorptance(t_0) parameter derived from the 5 minutes Pt deposited sample.

8. Histograms of α parameter and GLMM fit results for α, absorptance(t_0) and $t_{50\%}$ from TA kinetics before and after Pt deposition.

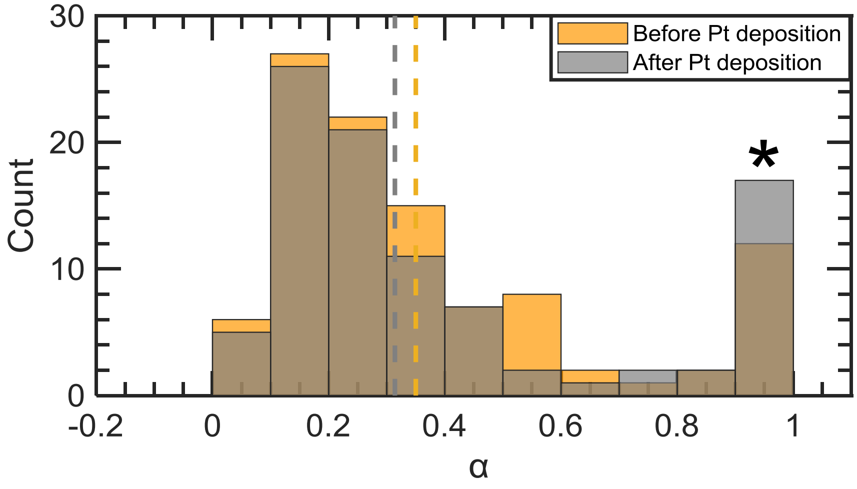

Figure S13. Histograms of parameter α derived from fits of the TA kinetic data from CN_x particles before and after 60 minutes of Pt deposition. * α=1 values have been excluded from the GLMM fitting due to overestimation of the parameter when the TA decays were fit.

Table S1. GLMM results: baseline values (before Pt) and predicted values (after Pt) of absorptance(t_0) and $t_{50\%}$ parameters for single (A and B) and all CN_x particles before and after 60 minutes of Pt deposition.

**Case**	**Parameter: $t_{50\%}$**				**Parameter: absorptance(t_0)**			
	**Before Pt deposition**		**After Pt deposition**		**Before Pt deposition**		**After Pt deposition**	
	**Baseline value**	**Error range (95% C.I.) of baseline value**	**Predicted value**	**Error range (95% C.I.) of the predicted value**	**Baseline value**	**Error range (95% C.I.) of the baseline value**	**Predicted value**	**Error range (95% C.I.) of the predicted value**
**Particle A**	53 μs	[37.1 μs, 75.1 μs]	309 μs	[188 μs, 508 μs]	0.0103	[0.0097, 0.0109]	0.0099	[0.0091, 0.0107]
**Particle B**	68 μs	[29.5 μs, 158.9 μs]	366 μs	[111.1 μs, 1.2 ms]	0.0048	[0.0042, 0.0054]	0.0043	[0.0036, 0.0051]
**All particles**	188 μs	[104.3 μs, 338 μs]	495 μs	[310 μs, 793 μs]	0.0051	[0.0038, 0.0068]	0.0044	[0.0040, 0.0048]

Table S2. GLMM results: baseline values (before Pt) and predicted values (after Pt) of the α parameter reported across all pixels from particles before and after 60 minutes of Pt deposition.

**Case**	**Parameter:** α			
	**Before Pt deposition**		**After Pt deposition**	
	**Baseline value**	**Error range (95% C.I.) of baseline value**	**Predicted value**	**Error range (95% C.I.) of the predicted value**
**All particles**	0.35	[0.30,0.41]	0.31	[0.27, 0.37]

9. Data from spatially correlated TAM measurements before and after 5 minutes of Pt deposition (6 particles)

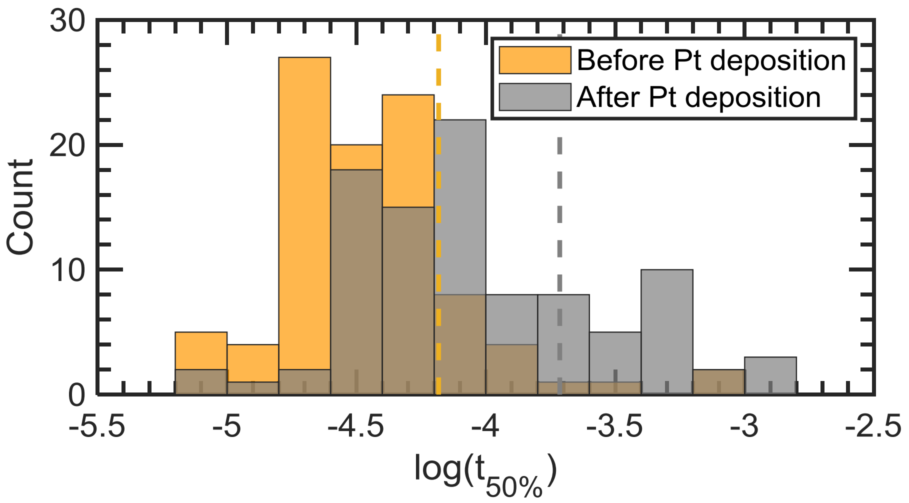

Figure S14. Histograms of log($t_{50\%}$) parameter derived from fits of the TA kinetic data from particles before and after 5 minutes of Pt deposition considering all pixels.

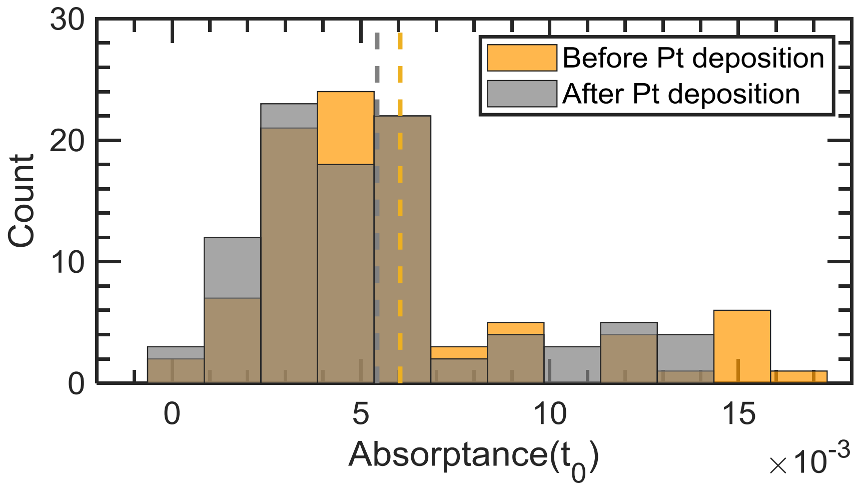

Figure S15. Histograms of absorptance(t_0) parameter derived from fits of the TA kinetic data from particles before and after 5 minutes of Pt deposition considering all pixels.

Table S3. GLMM results: baseline values (before Pt) and predicted values (after Pt) of the $t_{50\%}$ parameter reported across all pixels from particles before and after 5 minutes of Pt deposition.

**Case**	**Parameter: $t_{50\%}$**				**Parameter: absorptance(t_0)**			
	**Before Pt deposition**		**After Pt deposition**		**Before Pt deposition**		**After Pt deposition**	
	**Baseline value**	**Error range (95% C.I.) of baseline value**	**Predicted value**	**Error range (95% C.I.) of the predicted value**	**Baseline value**	**Error range (95% C.I.) of the baseline value**	**Predicted value**	**Error range (95% C.I.) of the predicted value**
**All particles**	66 µs	[41 µs, 105 µs]	193 µs	[128 µs, 290 µs]	0.0060	[0.0043, 0.0084]	0.0054	[0.0050, 0.0059]

Analysis was conducted on data from 6 spatially correlated maps (6 x 16 pixels) before and after 5 minutes of Pt deposition. The data was divided into four quartiles based on the $t_{50\%}$ data before Pt deposition. This approach allowed to assess the different ranges of $t_{50\%}$ (before Pt) to see if there was a selective influence of the deposition on specific subset of pixels with lower or higher $t_{50\%}$ values post Pt deposition. A similar GLMM approach as explained earlier was carrier out for quartile-wise $t_{50\%}$ values to calculate the parameters (baseline and predicted values) and quantify the relative change after Pt deposition. The percentage of pixels that show an increase have a clear downward trend with the increase in quartile. Relative increase considering all the pixels also indicate higher values for the lower quartiles, meaning the charges that the pixels with the lower initial $t_{50\%}$ tend to undergo the highest increase.

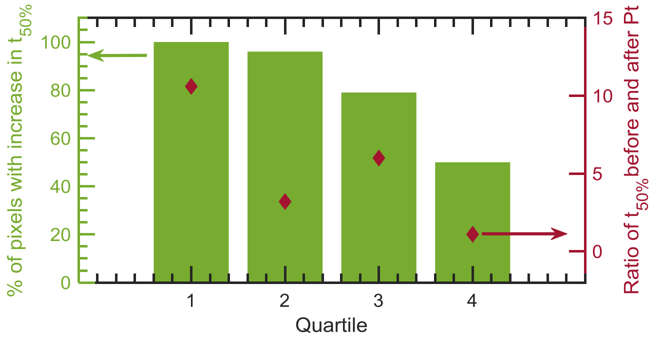

Figure S16. Quartile analysis showing the percentage of pixels that increased in $t_{50\%}$ values following 5 minutes of Pt deposition and the ratio between the baseline and predicted values, with respect to quartiles of the baseline values before Pt.

10. TA decay trace fitting and determination of the parameters

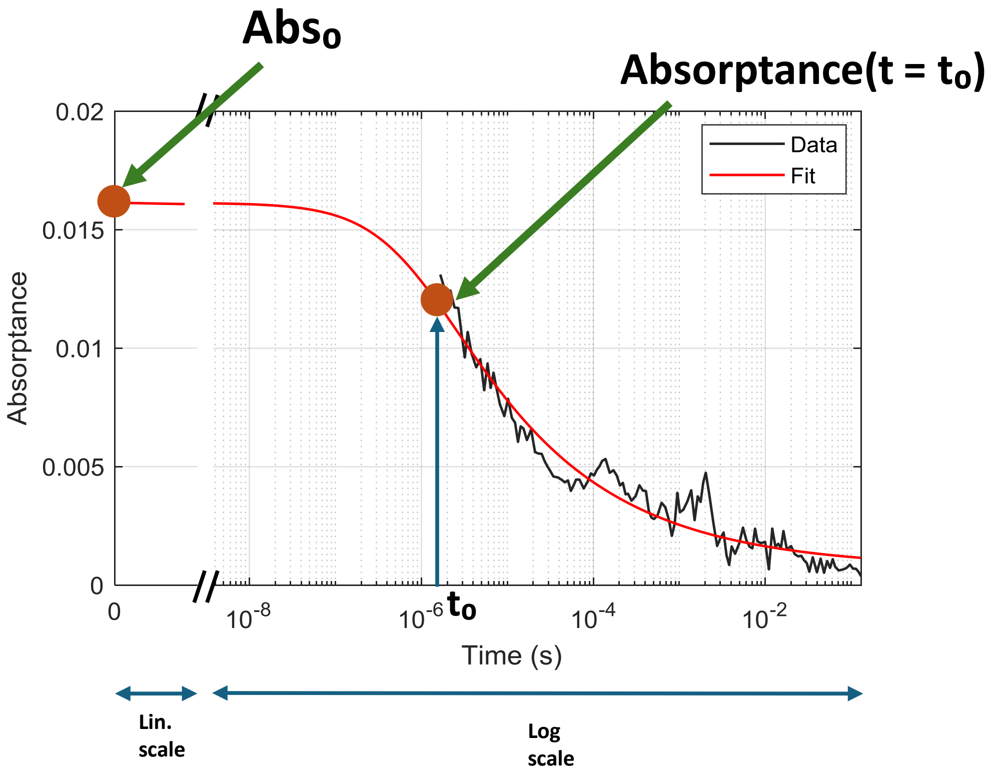

Figure S17. A sample TA decay trace depicting the difference between Abs_0 and Absorptance($t=t_0$).

The decay is fit using **Equation 1** of the main text. It is important to note that the Abs_0 is the absorptance extrapolated to time = 0, and the Absorptance($t = t_0$) is the absorptance value evaluated from the fit equation at the chosen t_0 of 1.5 μs (**Figure S17**). This Absorptance($t = t_0$) is more representative of the undecayed charge population for the $t_{50\%}$ calculation and is used as the Abs(t_0) parameter. The $t_{50\%}$ parameter is calculated from here by finding the time corresponding to the [Absorptance(t = 1.5 μs)/2] value from the fit line.

11. Correlation between $t_{50\%}$ vs. Absorptance(t_0) including all pixels of the TAM data collected from all bare CN_x particles (from the 60-minute Pt deposition data set)

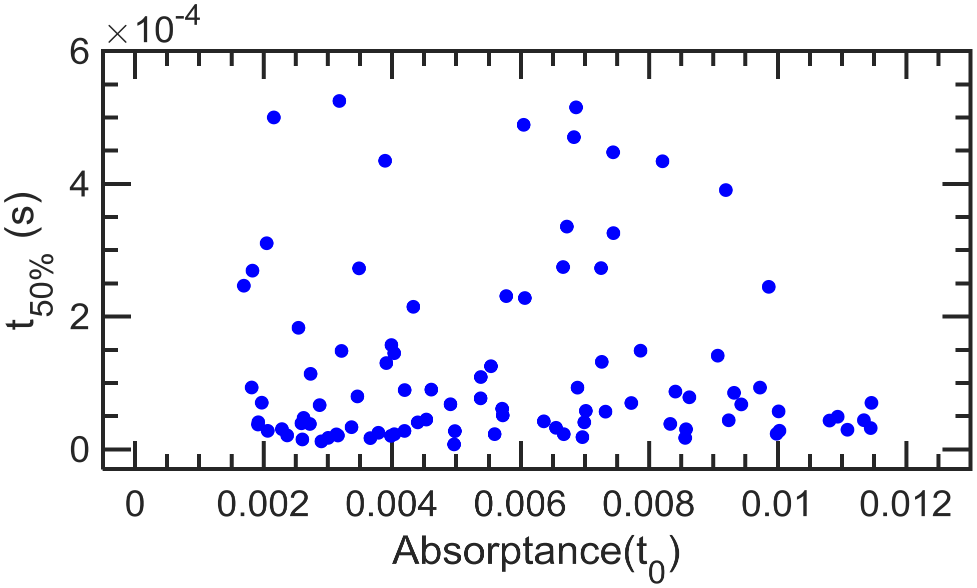

Figure S18. $t_{50\%}$ (s) vs. Absorptance(t_0) data from all CN_x particles (all pixels) measured before subjecting to 60 min Pt deposition.

References

[1] J. Pankratz, E. Mitchell, R. Godin, *Nanoscale* **2022**, *14*, 13580.
[2] C. Lu, J. Wu, D. Liu, *Materials Letters* **2018**, *227*, 308.
[3] D. J. Davidson, A. E. Martin, *Acta Psychologica* **2013**, *144*, 83.
[4] Z. Yu, M. Guindani, S. F. Grieco, L. Chen, T. C. Holmes, X. Xu, *Neuron* **2022**, *110*, 21.
